# Identification of Low-Nitrogen-Related miRNAs and Their Target Genes in Sugarcane and the Role of *miR156* in Nitrogen Assimilation

**DOI:** 10.3390/ijms232113187

**Published:** 2022-10-29

**Authors:** Shiwu Gao, Yingying Yang, Yuting Yang, Xu Zhang, Yachun Su, Jinlong Guo, Youxiong Que, Liping Xu

**Affiliations:** 1 Key Laboratory of Sugarcane Biology and Genetic Breeding, Ministry of Agriculture and Rural Affairs, National Engineering Research Center for Sugarcane, Fujian Agriculture and Forestry University, Fuzhou 350002, China; 2 Fujian Universities and Colleges Engineering Research Center of Modern Facility Agriculture, School of Food and Bioengineering, Fujian Polytechnic Normal University, Fuqing 350300, China; 3 Swammerdam Institute for Life Sciences, University of Amsterdam, 1098 XH Amsterdam, The Netherlands

**Keywords:** low nitrogen, sugarcane, root miRNA database, nitrogen use efficiency, *miR156*, overexpression

## Abstract

Chemical nitrogen (N) fertilizer is widely used in sugarcane production, especially in China and India. Understanding the molecular mechanisms and mining miRNAs and their target genes associated with nitrogen use efficiency (NUE) in sugarcane can aid in developing the N-efficient varieties, and thus is beneficial to reduce N fertilizer application. In this study, the root miRNA database of N-efficient sugarcane variety ROC22 under low N stress (0.3 mM NH_4_NO_3_) for 3 h was constructed, along with their transcriptome-rearranged data. KEGG analysis indicated that those candidate target genes, corresponding to differentially expressed miRNAs, were mainly enriched in N metabolism, amino acid metabolism, carbohydrate metabolism, photosynthesis, and hormone signal transduction pathways. It was found that under low N stress for 0–24 h, there was a negative correlation between *miR168* and *SPX*, along with *miR396* and *acnA*. Furthermore, the expression of *miR156* in the roots of ROC22 was significantly up-regulated under low N treatment. Compared with the wild-type, the *Arabidopsis* plants overexpressing sugarcane *miR156* exhibited significantly improved length and surface area of roots, while the expression of one NO_3_^−^ transporter gene *NRT1.1*, three N assimilation key genes (*NR1*, *NIR1*, and *GS*), and the activity of two N assimilation key enzymes (NR and GS) were up-regulated under low N treatment. It can be reasonably deduced that sugarcane *miR156* can enhance the nitrogen assimilation ability of the overexpressed *Arabidopsis* plants under low N application, and thus has a potential ability for improving sugarcane NUE. The present study should be helpful for understanding the molecular regulatory network in the N-efficient sugarcane genotype responding to low N stress and could provide the candidate miRNAs with a potential function in improving sugarcane NUE.

## 1. Introduction

N is an essential macro nutrient element for crops, and it is an important component of nucleic acid, phospholipid, and protein [1]. Sugarcane is the most important sugar crop [2,3]. N is one of the limiting factors for cane tonnage. Due to the limited arable land in China, sugarcane has been continuously cultivated for 20 to 30 years without rotation in most planting areas. To improve the yield, the N fertilizer application amount per sugarcane season was 400–800 kg/ha in China, which was much higher than that in other countries [4]. Excessive application of chemical N fertilizer has led to a cost increase, acidic soil, eutrophic water, and non-point source pollution, as well as the decrease in sucrose content in sugarcane [4,5]. It is gratifying that there are significant differences in NUE among different genotypes of sugarcane. The cultivation of N-efficient sugarcane varieties is an effective way to improve NUE and thus leads to a reduction in N application. The molecular mechanism of N uptake and utilization in plants is the basis of breeding N-efficient varieties.

In many biological metabolic processes, especially in stress response, miRNAs play an important regulatory role by inducing or inhibiting gene expression [3,6,7]. As reported, miRNAs play an important role in the regulation of plant nutrition [8,9,10]. Under low N stress, plants perceive the signal of N deficiency, amplify and transmit this signal to the responding molecules. Several miRNAs can cause mRNA degradation or translation inhibition of target genes, and regulate the expression of genes related to low N stress at the transcriptional and post transcriptional levels, thus open up the resistance mechanism in plants under low N stress [11].

Understanding the molecular response mechanism of crops under low N stress and analyzing the biological function of miRNA can lay a scientific foundation for improving crop NUE [8,12]. The miRNAs profiles and their regulatory mechanisms under low N stress were investigated in several kinds of plants, such as *Arabidopsis thaliana* [13], *Oryza sativa* [14], *Zea may* [9,15], *Coffea arabica* [16] and *Dendranthema morifolium* [12], etc.

*MiR156* plays an important role in plant growth and development [17,18] and in the adversity [8,12,19,20]. The expression of *miR156* could be induced by salt and drought in rice [21] and maize [22], and low temperature in rice [21]. The up-regulated *miR156* might have contributed to the growth of peanut roots under K deficiency [23]. The *miR156* could even regulate root regeneration and N fixation activity in alfalfa [24]. However, there has been no report of miRNA involvement in the regulatory response mechanism under low N stress in sugarcane until now. Therefore, the differentially expressed miRNAs and their candidate target genes associated with N utilization in sugarcane roots were analyzed based on the root miRNA in this study and the mRNA transcriptome databases [25] constructed from the N-efficient sugarcane variety ROC22 under low N treatment. In addition, the physiological and molecular regulation mechanism of the transgenic *Arabidopsis* plants overexpressing sugarcane *miR156* in low N treatment were investigated. This study aims to provide the candidate miRNAs with a potential function in improving sugarcane NUE, which should also help to understand the molecular regulatory network in sugarcane response to low N stress.

## 2. Results

### 2.1. sRNA Sequencing Data

The quality of sRNA sequencing samples met the requirements of library construction (class A), and the error rate of raw data from the six libraries constructed in this experiment was less than 0.02%, Q20 > 98.00% and Q30 > 97.50% (Table S1). The GC content was 52.00%. Clean reads were obtained by removing low-quality reads and reads with connectors from raw data (Table S2). The sequence of sRNA without annotation was the largest, followed by rRNA. The length distribution of sRNA sequences was shown in Figure S1. The clean sequence length of sugarcane sRNAs was generally 18–30 nt, and the peak value was concentrated at 24 nt which is similar to the peaks of 21 nt and 24 nt in plants [26]. These indicated that the quality of sequencing data was high.

### 2.2. Weighted Gene Co-Expression Network Construction and Module Identification

**Figure 1:**
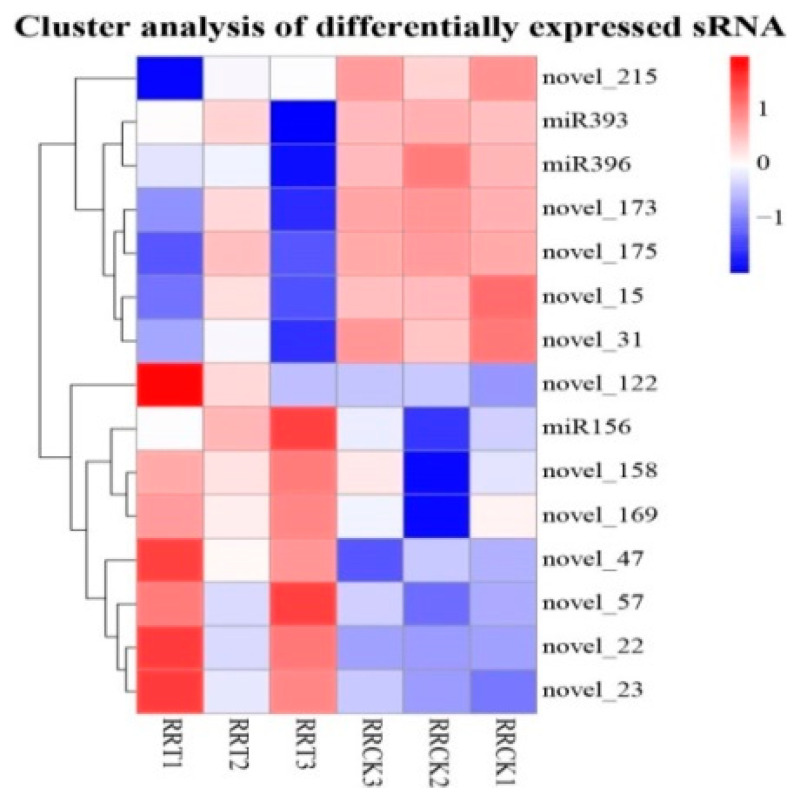
Hierarchical clustering map of the differentially expressed miRNAs. Notes: Clustering with log_10_ (TPM + 1) value, red indicated up-regulated miRNAs, and blue indicated down-regulated miRNAs. RRCK, the roots of ROC22 cultured in the low N solution (N source: 0.3 mM NH_4_NO_3_) for 0 h; RRT, the roots of ROC22 cultured in the low N solution for 3 h.

A total of 78 miRNAs were identified in the six libraries. There were 15 differentially expressed miRNAs in the roots of ROC22 under low N stress (RRT vs. RRCK), including three known miRNAs: *miR156* (significantly up-regulated), *miR396* (significantly down-regulated) and *miR393* (significantly down-regulated). Among the 12 differentially expressed new miRNAs, seven miRNAs (*novel-22*, *novel-23*, *novel-47*, *novel-57*, *novel-122*, *novel-158*, and *novel-169*) were up-regulated and the other five miRNAs (*novel-15*, *novel-31*, *novel-173*, *novel-175* and *novel-215*) were down-regulated. Figure 1 shows the hierarchical clustering results of the expression patterns of differentially expressed miRNAs.

### 2.3. Prediction and Function of Target Genes of miRNA

In plants, miRNAs participate in various life processes by regulating their target genes [9,11]. Based on the root transcriptome database of ROC22 under low N stress [25] and the sugarcane EST database (about 284,818 ESTs) in NCBI, 10,454 target genes were predicted. GO enrichment and KEGG analysis were then carried out by GOseq and KOBAS (2.0) software [27]. According to GO analysis (Figure S2), the 2056 predicted target genes of differentially expressed miRNAs were involved in the biological process, cellular component, and molecular function. Among them, the predicted target genes were mainly involved in the molecular functions of starch synthase, mannosyltransferase, asparagine hydrolase, and other biological processes, such as abiotic stimulation and hormone response. KEGG analysis showed that the predicted target genes were mainly concentrated in RNA transport, N metabolism, carbohydrate metabolism (starch and sucrose metabolism), amino acid metabolism (cysteine and methionine metabolism; glycine, serine and threonine metabolism; valine, leucine and isoleucine degradation) and hormone signal transduction pathways.

### 2.4. Crosstalk with miRNA and mRNA Transcriptome Sequencing Data

**Figure 2:**
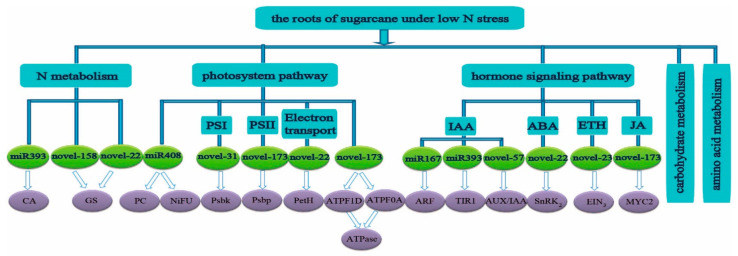
The main enrichment pathways of the candidate genes corresponding to the differentially expressed miRNAs in sugarcane roots under low N stress (N source: 0.3 mM NH_4_NO_3_). *CA*, carbonic anhydrases gene; *GS*, glutamine synthetase gene; *PC*, plastocyanin gene; *NiFU*, NiFU-like domain-containing protein gene; *Psbk*, photosystem I subunit X gene; *Psbp*, photosystem II oxygen-evolving enhancer protein gene; *PetH*, ferredoxin-NADP^+^ reductase gene; *ATPF1D*, a subunit of mitochondrial ATP synthase gene; *ATPF0A*, F-type H^+^-transporting ATPase subunit gamma gene; *ARF*, auxin response factor gene; *AUX/IAA*, auxin/indole-3-acetic acid gene; *TIR1*, transport inhibitor response gene; *SnRK2*, SNF1-related protein kinase gene; *EIN3*, ethylene-insensitive gene; *MYC2*, myelocytomatosis protein gene.

Those target genes in the roots were mainly concentrated in the N metabolism, amino acid metabolism, carbohydrate metabolism, photosynthesis, and hormone signal transduction pathways under low N stress (Figure 2). In the N metabolism pathway, *novel-158* and *novel-22* might target *GS*, and *miR393* might target *CA*, all participated in the N assimilation process. In the plant hormone signal transduction pathway, *miR167* targeted auxin response factor (*ARF*), and *miR393* might target transport inhibitor response 1 (*TIR1*), both participate in the IAA pathway, *novel-22* might target *SnRK_2_* to participate in the ABA signaling pathway; *novel-23* might participate in ETH pathway through targeting *EIN_3_*, and *novel-173* might participate in JA signaling pathway through targeting *MYC2*.

**Figure 3:**
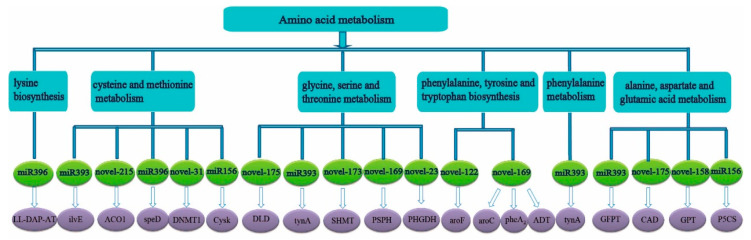
Amino acid metabolic pathway map exhibiting an enrichment of predicted target genes corresponding to the differential miRNAs in sugarcane roots under low N stress. Note: *LL-DAP-AT*, LL-diaminopimelate aminotransferase gene; *ilvE*, branched-chain amino acid aminotransferase gene; *ACO1*, aminocyclopropane carboxylate oxidase gene; *speD*, S-adenosylmethionine decarboxylase gene; *DNMT1*, DNA (cytosine-5)-methyltransferase gene; *Cysk*, cysteine synthase gene; *DLD*, dihydrolipoamide dehydrogenase gene; *tynA*, primary-amine oxidase gene; *SHMT*, glycine hydroxymethyltransferase gene; *PSPH*, phosphoserine phosphatase gene; *PHGDH*, D-3-phosphoglycerate dehydrogenase gene; *aroF*, 3-deoxy-7-phosphoheptulonate synthase gene; *aroC*, chorismate synthase gene; *pheA_2_*, prephenate dehydratase gene; *ADT*, arogenate/prephenate dehydratase gene; *GFPT*, glucosamine-fructose-6-phosphate aminotransferase gene; *CAD*, aspartate carbamoyltransferase gene; *GPT*, alanine transaminase gene; *P5CS*, 1-pyrroline-5-carboxylate dehydrogenase gene.

**Figure 4:**
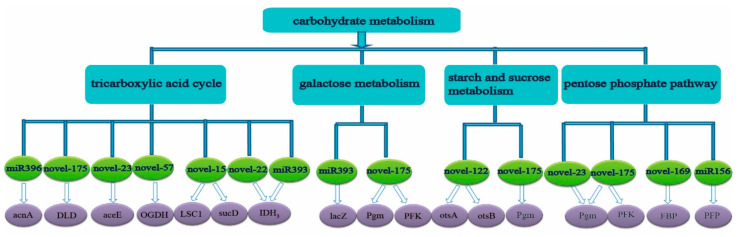
The carbohydrate metabolic pathway map of predicted target gene enrichment corresponding to differential miRNAs in sugarcane roots under low N Stress (N source: 0.3 mM NH_4_NO_3_) Note: *acnA*, aconitate hydratase gene; *DLD*, dihydrolipoamide dehydrogenase gene; *ace*, pyruvate dehydrogenase E1 component gene; *OGDH*, 2-oxoglutarate dehydrogenase E1 component gene; *LSC1*, succinyl-CoA synthetase alpha subunit gene; *sucD*, succinyl-CoA synthetase alpha subunit gene; *IDH3*, isocitrate dehydrogenase (NAD^+^) gene; *lacZ*, beta-galactosidase gene; *Pgm*, phosphoglucomutase gene; *PFK*, 6-phosphofructokinase gene; *otsA*, trehalose 6-phosphate synthase gene; *otsB*, trehalose 6-phosphate phosphatase gene; *FBP*, fructose-1,6-bisphosphatase I gene; *PFP*, diphosphate-dependent phosphofructokinase gene.

The predicted target genes of miRNAs in the roots were also concentrated in alanine, aspartate and glutamic acid metabolism, phenylalanine metabolism, phenylalanine, tyrosine and tryptophan biosynthesis, glycine, serine and threonine metabolism, cysteine and methionine metabolism, lysine biosynthesis (Figure 3) and the tricarboxylic acid cycle, galactose metabolism, starch and sucrose metabolism, pentose phosphate pathway in carbohydrate metabolism (Figure 4).

In addition, some predicted target genes in the roots were also related to the photosynthetic pathway. Among them, *novel-173* might participate in the photosynthetic pathway by targeting *PsbP* and *ATPase* (*ATPF1D* and *ATPF0A*), and *novel-22* might participate in the electron transport in photosynthesis by targeting *PetH*, while *novel-31* might participate in the photosystem I pathway by targeting *Psbk*, and *miR408* might participate in the pathway of the photosystem by targeting plastocyanin (*PC*) and *NiFU* (Figure 2). Among the identified miRNAs, the squamosa promoter binding protein-like gene (*SPL*, sample|Cluster-9046.200116), which was assumed to be the predicted target gene of *miR156* in the roots of ROC22, was down-regulated by 0.61-fold at 3 h under low N stress. *miR156* might also participate in the metabolism of alanine, aspartate and glutamate by targeting 1-pyrroline-5-carboxylate dehydrogenase gene (*P5CS*, sample|Cluster-9046.62773, log_2_ FC = −1.4) (Figure 3). In the roots of ROC22, *GRF* (growth regulating factor, sample|cluster-9046.236180), the predicted target gene of *miR396*, was up-regulated by 2.48-fold. *miR396* might also participate in the tricarboxylic acid cycle by targeting aconitate hydratase (*acnA*, ncbi|Cluster-1234.10611, log_2_ FC= 0.46) (Figure 4). The predicted target gene of *miR408*, plastocyanin (*PC*, sample|cluster-9046.239128) was up-regulated by 5.39-fold (Figure 2). *miR393* might participate in IAA pathway by targeting *TIR1* (sample|cluster-82409.0, log_2_ FC = −0.50) (Figure 2).

Among the identified unknown miRNAs, *novel-158* might participate in N assimilation by targeting *GS* (sample|cluster-9046.93347, log_2_ FC = −2.019) (Figure 2). *Novel-57* might participate in plant hormone signal transduction pathway-IAA pathway by targeting *AUX/IAA* (sample|Cluster-9046.46359, log_2_ FC = −1.5) (Figure 2). *Novel-215* might participate in amino acid metabolism by targeting cysteine and methionine metabolism-aminocyclopropane carboxylate oxidase (*ACO1*, sample|Cluster-9046.137637, log_2_ FC = 1.80) (Figure 3).

### 2.5. Verification of the Transcriptome Sequencing Data of miRNAs and Candidate Target Genes by qRT-PCR

**Figure 5:**
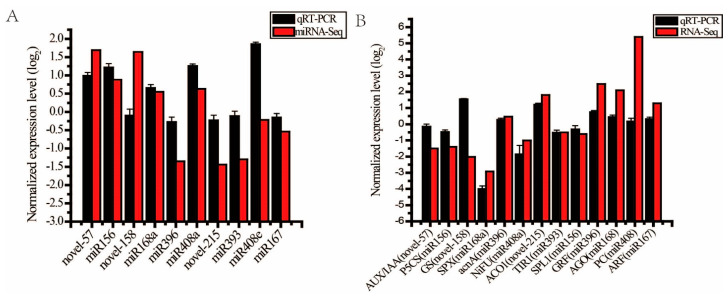
The qRT-PCR validation of the expression of miRNAs and its candidate target genes. Note: (**A**) The qRT-PCR and miRNA-Seq results of ten miRNA. (**B**) The qRT-PCR and RNA-Seq results of 13 candidate target genes. *AUX/IAA*, auxin/indole-3-acetic acid gene; *P5CS*, 1-pyrroline-5-carboxylate dehydrogenase gene; *GS*, glutamine synthase genes gene; *SPX*, SPX domain-containing protein gene; acnA, aconitate hydratase gene; *NiFU*, NiFU-like domain-containing protein gene; *ACO1*, aminocyclopropane carboxylate oxidase gene; *TIR1*, transport inhibitor response gene; *SPL1*, squamosa promoter binding protein-like gene; *GRF*, growth-regulating factor gene; *AGO*, argonaute gene; *PC*, plastocyanin gene; *ARF*, auxin response factor gene.

Ten miRNAs and 13 candidate target genes were randomly selected for qRT-PCR expression validation. As shown in Figure 5A,B, the qRT-PCR results of miRNA and candidate target genes were basically consistent with the miRNA sequencing data (miRNA-Seq) and transcriptome sequencing data (RNA-Seq), but the degree of up-regulation or down-regulation was different from the transcriptome, which might be due to the reason that sequencing is more sensitive than qRT-PCR detection [28].

### 2.6. Expression Trend of miRNA and Candidate Target Genes in Sugarcane under Low N Stress

**Figure 6:**
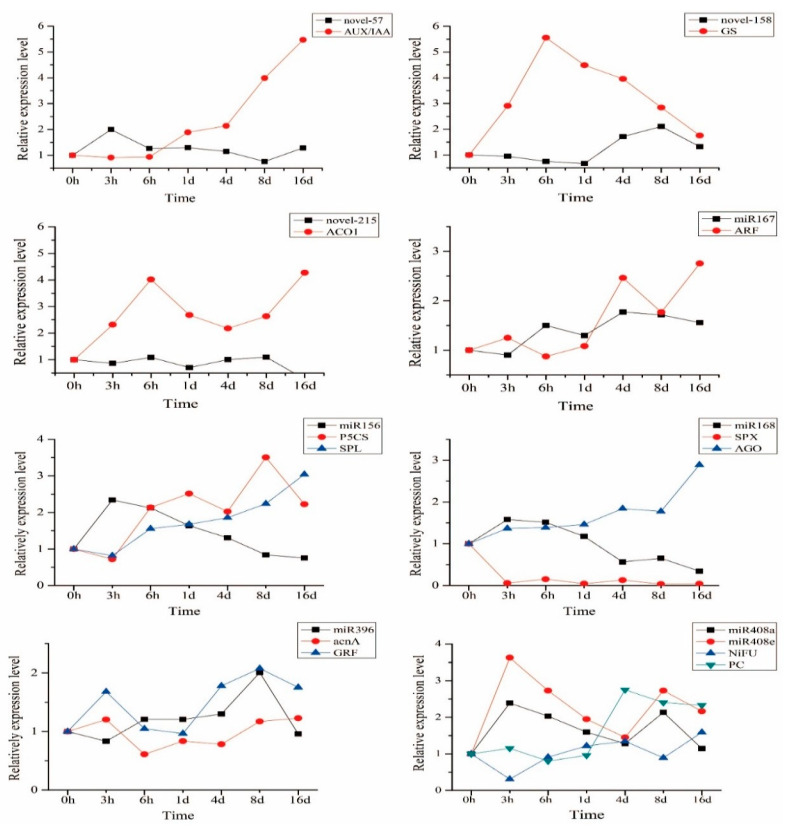
Expression trends of eight miRNAs and 12 candidate target genes in the roots of sugarcane ROC22 under low N stress (N source: 0.3 mM NH_4_NO_3_). Note: *AUX/IAA*, auxin/indole-3-acetic acid gene; *GS*, glutamine synthase gene; *ACO1*, aminocyclopropane carboxylate oxidase gene; *ARF*, auxin response factor gene; *P5CS*, 1-pyrroline-5-carboxylate dehydrogenase gene; *SPL1*, squamosa promoter binding protein-like gene; *SPX*, SPX domain-containing protein gene; *AGO*, argonaute gene; acnA, aconitate hydratase gene; *GRF*, growth-regulating factor gene; *NiFU*, NiFU-like domain-containing protein gene; *PC*, plastocyanin gene.

According to the negative correlation between the expression of the miRNAs and their predicted corresponding target genes in the sequencing data, eight miRNAs and 12 candidate target genes were selected and detected by qRT-PCR in the roots of ROC22 at 0–16 d under low N stress (Figure 6). Under low N stress for 0 h–1 d, *novel-57* was negatively correlated with *AUX/IAA*, and similar for *novel-158* and *GS*, *miR167* and *ARF*, respectively. Under low N stress for 0–16 d, there was a negative correlation between *novel-215* and *ACO1*. Under low N stress for 3 h, 8 d and 16 d, *miR156* was negatively correlated with *SPL* and *P5CS*. *miR168* was negatively correlated with *SPX* and *AGO* at 0–1 d and 1–16 d under low N stress, respectively. The negative correlation between *miR396* and *acnA* was obvious under stress for 0 h–1 d, and so was between *NiFU* and *miR408a*/*miR408e* for 0 h–6 h.

Compared with the control, the expression level of *miR156* was significantly up-regulated by 2.3-, 2.1- and 1.6-fold in the roots of ROC22 under low N stress for 3 h, 6 h and 1 d, respectively (Figure 6). *miR156* may be one of the key genes in sugarcane under low N stress. Considering that the genetic transformation efficiency of sugarcane was extremely low and unstable, the physiological and molecular evaluation results of the gene in different plants could also help to understand the biological function of the gene. Therefore, we transformed sugarcane *miR156* into *Arabidopsis*, which laid the foundation for the subsequent identification of the gene function in sugarcane.

### 2.7. Characterization of the miR156-Overexpressed Arabidopsis Plants under Low N Treatment

**Figure 7:**
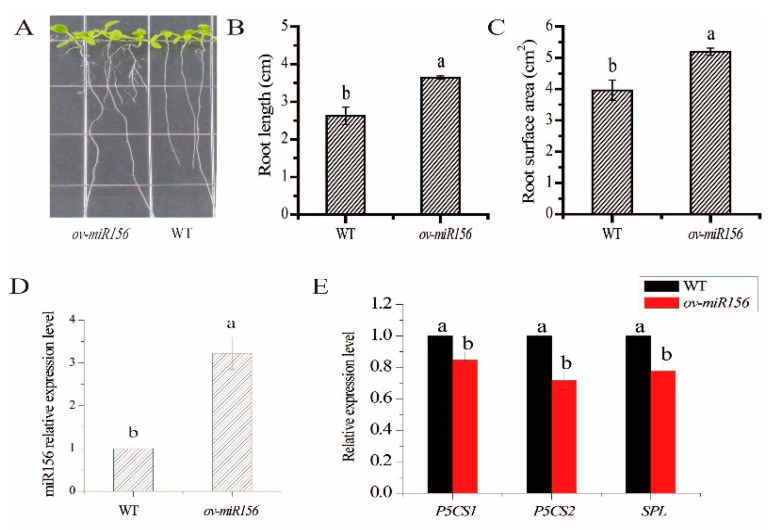
Root phenotype, root phenotype parameters, and the expression of *miR156* and its predicted target genes in the *Arabidopsis* plants overexpressing sugarcane *miR156* under low N treatment. Note: (**A**) The root phenotype in transgenic *Arabidopsis* overexpressing *miR156* under low N treatment. (**B**) The root length in transgenic *Arabidopsis* overexpressing *miR156* under low N treatment. (**C**) The root surface area in transgenic *Arabidopsis* overexpressing *miR156* under low N treatment. (**D**) The relative expression of *miR156* in the *Arabidopsis* overexpressing *miR156* under low N treatment. (**E**) The relative genes expression of *P5CS1*, *P5CS2*, and *SPL* in the *Arabidopsis* overexpressing *miR156* under low N treatment. WT: wild type rice; *ov-miR156*: the *Arabidopsis* overexpressing *miR156*. *P5CS*, 1-pyrroline-5- carboxylate dehydrogenase gene; *SPL*, squamosa promoter binding protein-like gene. LN: Low N treatment. Data are mean ± standard error of three replicates. Bars with different letters are significantly different at the *p* < 0.05 level.

Root phenotypic characteristics of the *ov-miR156* transgenic and wild type *Arabidopsis* under low N treatment were compared in Figure 7A. The length and surface area of roots in the *miR156-*overexpressed *Arabidopsis* plants were 1.39- and 1.31-fold longer than those of wild type under low N treatment (Figure 7B,C). Compared with the wild type, the expression level of *miR156* was significantly up-regulated in the transgenic plants under low N treatment (Figure 7D). Moreover, three target genes of *miR156*, i.e., *SPL*, *P5CS1*, and *P5CS2*, all showed a significant downward trend simultaneously (Figure 7E).

### 2.8. Changes in the N Assimilation Key Genes Expressions and Enzymes Activities in the miR156-Overexpressed Arabidopsis under Low N Treatment

**Figure 8:**
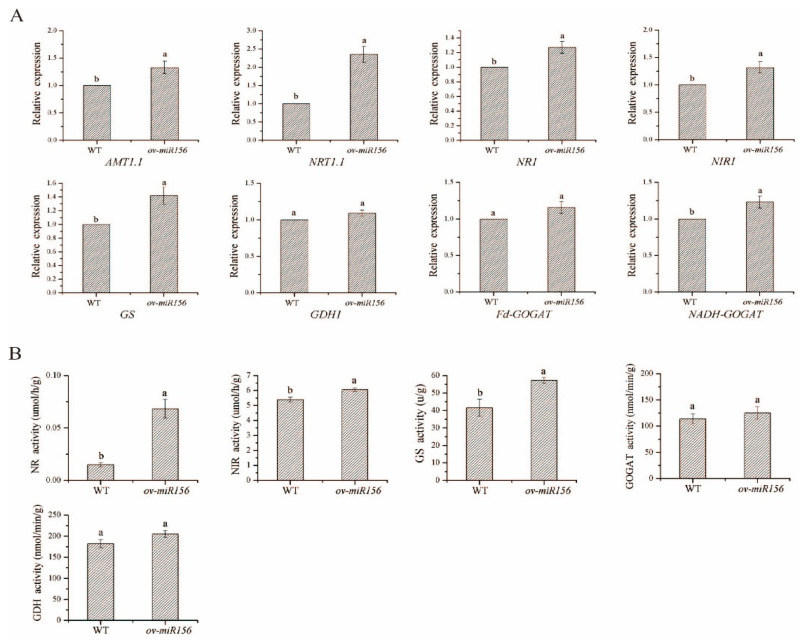
The expression levels of key genes and assimilation enzyme activities in N metabolism in transgenic *Arabidopsis* overexpressing *miR156* under low N treatment. Note: (**A**) The expression levels of N metabolism key genes in transgenic *Arabidopsis* overexpressing *miR156*. (**B**) The activities of N metabolism assimilation enzymes in transgenic *Arabidopsis* overexpressing *miR156*. WT: wild type rice; *ov-miR156*: transgenic *Arabidopsis* overexpressing *miR156*. Data are mean ± standard error of three replicates. Bars with different letters are significantly different at the *p* < 0.05 level.

At the molecular level, the expression levels of ammonium transporter gene *AMT1.1*, nitrate transporter gene *NRT1.1*, N assimilation key genes *NR1, NIR1, GS* and *NADH-GOGAT* in the *miR156*-overexpressed *Arabidopsis* were significantly higher than those in the wild-type plants under low N stress (Figure 8A).

Under low N treatment, its activity of the NR enzyme in the *miR156*-overexpressed plants, which limited N primary assimilation rate, was significantly increased and was 3.56-fold higher than that of the wild type. Additionally, the enzyme activities of NIR and GS were 12.30% and 38.03% higher than those of the wild type, respectively (Figure 8B).

## 3. Discussion

### 3.1. The miRNA Data of Sugarcane Root under Low N Stress Were Reliable

miRNA plays an important role in the molecular regulatory network of crop response to low N stress [29,30]. Vidal et al. [13] found 40 nitrate-responsive genes and novel miRNAs actively respond to nitrate changes by RNA-seq and sRNA-seq in the roots of *Arabidopsis* under different N treatments, such as *miR5640* and its predicted target gene *AtPPC3*. After analyzing the expression trends of IncRNAs, miRNAs and predicted target genes in rice under N deficiency, Shin et al. [14] found the targeting relationship between root-specific *miR444a4-3p* and *MADS25*. Santos et al. [16] identified 86 miRNAs and 253 predicted target genes in coffee under low N conditions. In this study, 78 miRNAs were identified in sugarcane under low N stress. The peak of sequence length (24 nt) of sRNA in this study is comparable to them in plants (generally 21 nt and 24 nt) [26].

### 3.2. The Predicted Target Genes of Differentially Expressed miRNA Were Mainly Concentrated in N Metabolism, Carbohydrate Metabolism, and Amino Acid Metabolism

miRNA regulates the target genes’ expression by binding with 3’UTR of the target gene, inhibiting or degrading mRNA translation [31]. The most direct way to explore the regulation mechanism of miRNA is to study the biological functions of its targeted genes [32], and bioinformatics analysis can better predict the target genes of miRNA [33]. In order to explore the regulatory function of differentially expressed miRNAs, the potential target genes of miRNAs were predicted by using the mRNA transcriptome database [25] of ROC22 root under low N stress.

In the KEGG analysis of predicted target genes of differentially expressed miRNA in Soybean under low N stress, it was found that these genes were involved in protein degradation, N metabolism, carbon metabolism, amino acid metabolism, hormone signaling pathway, cell transport and other metabolic pathways and regulatory pathways [29]. The predicted target genes of differentially expressed miRNAs in the root of *Arabidopsis* under different nitrate treatments were mainly concentrated in carbon (C) and N metabolism and amino acid metabolism [13], which was similar to what we found (Figure 1). Here, under low N stress, the expression levels of key genes of N metabolism and C metabolism in sugarcane were significantly changed. In the process of the absorption of nitrate and ammonium, nitrate assimilation and biological synthesis of N-containing macromolecules required a lot of energy provided by C metabolism [29]. In addition, under low N stress, the predicted target genes of differentially expressed miRNAs were more concentrated in amino acid metabolism.

### 3.3. miRNA Was Differentially Expressed in Sugarcane under Low N Stress

Wang et al. [8] found that *miR156* and *miR396* responded positively to environmental stress in the new leaves of poplar cuttings under N deficiency. Under N starvation stress, the expression of *miR156* was up-regulated in *Arabidopsis* seedlings [34]. The expression of *miR156* was up-regulated in the roots of Chrysanthemum under low N stress [12]. Under low N stress (0.02 mM N), the expression of *miR396* was up-regulated in poplar seedlings [20]. In this research, sugarcane *miR156* was up-regulated and *miR396* was down-regulated under low N stress (0.06 mM N) for 3 h. The expression trend of the sugarcane *miR396* was not consistent with that reported in poplar [20], which might be due to various action patterns of miRNA in different crops.

The expression of *miR408* was down-regulated in *Arabidopsis* seedlings under N starvation stress [34]. Under short-term (0.5 h, 2 h, 6 h and 12 h) low N stress (0.75 mM N), the expression of *miR408* was down-regulated in the roots of soybean varieties with low N adaptability and up-regulated under long-term low N stress. However, *miR167* and *miR168* did not respond to either short-term or long-term stress [29]. The expression of *miR159*, *miR167* and *miR168* was down-regulated in *Populus* seedlings under low N stress (0.02 mM N) [20]. In this research, the different expressions of *miR159*, *miR167*, *miR168* and *miR408* compared with the control were identified in the roots of ROC22 under low N stress, while without significance.

### 3.4. Diverse Expression Trends of miRNA and Predicted Target Genes in Sugarcane under Low N Stress

miRNAs regulate crop growth and development by acting on target genes and play an important role in the molecular regulatory network under low N stress [9]. For example, the expression levels of key miRNAs change significantly, which can inhibit or induce the expression of target genes, thus affecting the growth and development of crops [9,11]. In our sequencing database, the identified predicted target genes of miRNA had different biological functions, which could be divided into the following three categories.

The first was transcription factors. According to the literature reports, the target genes of *miRl56* and *miR396* were *SPL* [35,36] and *GRF* [8], respectively. Overexpressing *miR156* significantly enhanced root regeneration and N fixation by regulating *SPL* in *Medicago sativa* [24]. Here, the target gene *SPL* of differentially expressed *miR156* was down-regulated by 0.61-fold in the roots of ROC22 under low N stress for 3 h. Wang et al. [8] found that *miR396* responded positively to N deficiency by targeting *GRF* in poplar. In this study, *miR396* was significantly down-regulated (log_2_ FC = −1.35, pval = 0.02) and its target gene *GRF* was up-regulated by 2.48-fold in the roots of ROC22 under low N stress for 3 h. The target gene of *miR159* is *MYB* [37,38]. *MYB* was the most differentially expressed transcription factor in the roots of ROC22 under low N stress [25]. In the regulatory networks, transcription factors belong to upstream genes, which can combine with *cis* acting elements of downstream genes to regulate their expression [39]. miRNA targeted transcription factors might be involved in the regulation of some downstream genes in response to low N stress in sugarcane.

The second type was genes related to the hormone signal transduction pathway. miRNAs play an important regulatory role in the plant hormone signaling pathway and are induced by plant endogenous hormones. The target gene of *miRl67* was *ARF*, a response factor of auxin in the plant hormone signal transduction pathway [40]. It played an important regulatory role in the growth and development of the leaves and roots and flower organ development of plants [41]. *ARF* also responded positively to NO_3_^−^ signal, adapted to a low N environment, and regulated the structure and growth of lateral roots [42]. In this study, *miR167* was down-regulated by 0.54-fold and *ARF* was up-regulated by 1.29-fold under low N stress.

The third was enzyme protein genes. The target genes of *miR408* were *PC* [38] and *laccase* [43]. The *PC* gene was up-regulated by 5.39-fold in the roots of ROC22 under low N stress for 3 h, which positively responded to low N stress. The target gene of *miR168* was *AGO* [38]. *AGO* was up-regulated by 2.1-fold in the roots of ROC22 under low N stress for 3 h. These proteins belonged to enzyme coding genes and might play an important role in response to low N stress in sugarcane.

In addition, some of the target genes predicted by novel miRNA also had clear biological functions, while some had unclear biological functions. We speculated that the up-regulated expression of miRNA might lead to the degradation of target genes under low N stress and the down-regulated expression of miRNA might promote the overexpression of target genes, thus changing some metabolic pathways and signal transduction pathways and affecting the growth and development of sugarcane. The metabolic and signaling pathways regulated by miRNA and its predicted target genes would provide a new research direction for the molecular regulation mechanism of sugarcane under low N stress.

### 3.5. The miR156 from Sugarcane Roots Could Enhance the N Assimilation Ability of Arabidopsis under Low N Application

Exploring the targeting relationship between miRNA and its target genes could further understand the molecular regulatory mechanism of miRNA [8,20]. *miR156* had been extensively studied in response to abiotic stress [8,12,19,20,21,22], but the function of *miR156* in N assimilation has not been studied, including its role in N assimilation in *Arabidopsis*.

The relationship between *miR156* and its target gene *SPL* was studied in *Arabidopsis* [44], rice [21], and wheat [45]. Previous studies had found that *OsmiR156* might change the morphogenesis and grain size of rice by down-regulating the target gene *OsSPL*, thereby increasing the rice yield [46,47]. In *Arabidopsis* seedlings, *miR156* participated in the leaf development [44] and flowering process by regulating the target gene *SPL* [48]. In switchgrass, *miR156* regulated the apical dominance and flowering status by inhibiting the *SPL* gene [49]. In addition, *miR156* responded positively to salt stress by down regulating *SPL* family genes and key genes responding to salt stress [50]. These results indicated that *miR156* regulated plant growth and development by targeting *SPL*. In this research, compared with wild-type *Arabidopsis*, the *SPL* gene of transgenic *Arabidopsis* overexpressing *miR156* was significantly down-regulated under low N treatment, which verified the target relationship between *miR156* and *SPL*. Under low N treatment, the expression of *P5CS1* and *P5CS2*, a candidate target gene of *miR156*, was down-regulated, showing a targeted relationship with *miR156*. We speculated that sugarcane *miR156* might respond positively to low N treatment by negatively regulating *SPL*, *P5CS1*, and *P5CS2*. In future research, the targeting relationship between sugarcane *miR156* and candidate target genes *P5CS1* and *P5CS2* needed to be further verified by other experimental means.

Transgenic plants overexpressing *miR156* were often used as experimental materials to investigate the role of *miR156* in N stress [18,51]. Gou et al. [51] found that *miR156* promoted anthocyanin accumulation by regulating *SPL9* in transgenic *Arabidopsis* overexpressing *miR156* under N deficiency stress. In order to adapt to the low N stress environment, transgenic rice overexpressing *miR156* under low N stress down-regulated the key genes *CYP724* and *CYP90* in the brassinosteroid biosynthesis pathway [18]. In this research, the root length and root surface area of transgenic *Arabidopsis* overexpressing *miR156* were significantly higher than those of wild type under low N treatment. Nitrate which was absorbed by the plant roots, which was converted into ammonium salt by NR and NIR enzyme, and then converted into glutamine (Gln) and glutamate (Glu) mainly through two reaction pathways, one of which was GDH, and the other two were GS and GOGAT as the catalytic enzymes [52,53]. Gln and Glu, which served as precursors of N compound biosynthesis, were further converted into organic N molecules [54]. Therefore, NR, NIR, GS, GDH, and GOGAT were the key enzymes in the process of primary N assimilation. Furthermore, the expression of *NRT1.1*, *NR1*, *NIR1*, and *GS* of transgenic *Arabidopsis* overexpressing *miR156* were significantly higher than those of wild type under low N treatment. According to the root phenotype, physiological indexes, and the expression of key N assimilation genes, sugarcane *miR156* could enhance the N assimilation ability of *Arabidopsis* under low N application.

## 4. Materials and Methods

### 4.1. Cultivation of Plant Materials

Tissue cultured plantlets of ROC22 (low N tolerant variety) [55] were provided by the Key Laboratory of Sugarcane Biology and Genetic Breeding, Ministry of Agriculture and Rural Affairs, Fujian Agriculture and Forestry University (Fuzhou, Fujian, China). The ROC22 plantlets of uniform size were cultured with the modified Hoagland solution at 30 °C/25 °C (day/night) under a 16 h photoperiod (70% relative humidity). The elements in the hydroponic solution (pH6.0) were as follows: 3.0 mM NH_4_NO_3_, 1.0 mM KH_2_PO_4_, 2.0 mM KCl, 2.5 mM CaCl_2_·2H_2_O, 1.0 mM MgSO_4_·7H_2_O, 5.0 μM MnCl_2_·4H_2_O, 0.025 mM Fe-EDTA, 20.0 μM H_3_BO_3_, 0.4 μM ZnSO_4_·7H_2_O, 0.2 μM CuSO_4_·5H_2_O, and 0.05 μM Na_2_MoO_4_·2H_2_O. The hydroponic solution was refreshed every three days.

### 4.2. cDNA Library Construction and Small RNA (sRNA) Sequencing

The roots of four-leaf stage ROC22 plantlets of uniform size at 0 h and 3 h under low N treatment were collected. We set up 0.6 mM N concentration (N source: 0.3 mM NH_4_NO_3_) for 3 h as low N treatment and for 0 h as control (N source: 3.0 mM NH_4_NO_3_) [25]. The treatment and control were designated as RRCK and RRT. Three randomly selected plantlets were pooled into biological duplicates, and each experiment was performed using three biological replicates. The total RNA of samples was extracted with TRIzol reagent (Invitrogen, Shanghai, China), and RNA quality was examined using a NanoDrop (Thermo Fisher Scientific, Inc., Waltham, MA, USA) and an Agilent 2100 Bioanalyzer (Agilent Technologies, Santa Clara, CA, USA). The RNA was divided into two parts, one was sent to Novogene Corporation (Tianjin, China) for miRNAsequencing, and the other for qRT-PCR validation analysis.

The analysis method of miRNAsequencing data was in reference to Yang et al. [27]. DESeq2 was used to analyze the difference of miRNA expression level. According to the *p* value of miRNA (*p* < 0.05), the significant level of miRNA was screened. The software of psRNATarget was used to predict the target genes of miRNA. Then, GOseq [27] was used to analyze the candidate target genes of differentially expressed miRNAs, and the functional differences of the genes between different samples were obtained. Finally, a hypergeometric test was used to analyze the KEGG enrichment of candidate target genes with differential expression of miRNAs, and to explore the biochemical metabolism and signal transduction pathway of candidate target genes.

### 4.3. Detection of miRNA and Target Gene Expression under Low N Stress

Ten miRNAs (*novel-57*, *miR156*, *novel-158*, *miR168a*, *miR396*, *miR408a*, *novel-215*, *miR393*, *miR408e*, *miR167*) and 13 candidate target genes (*AUX/IAA*, *P5CS*, *GS*, *SPX*, *acnA*, *NiFU*, *ACO1*, *TIR1*, *SPL1*, *GRF*, *AGO*, *PC*, *ARF*) were randomly selected, and their gene expression levels were validated by qRT-PCR. The primer sequences were listed in Tables S3 and S4. The genes of *18S rRNA* [56] and glyceraldehyde-3-phosphate dehydrogenase, *GAPDH* [57] were used as the internal reference genes. ROC22 plantlets at a four-leaf stage were used for low N treatment, and the samples were collected for the follow-on experiments at 0 h, 3 h, 6 h, 1 d, 4 d, 8 d and 16 d after treatment. We set up 0.6 mM N concentration (N source: 0.3 mM NH_4_NO_3_) as low N treatment, and the sample collected at 0 h as control.

### 4.4. Acquisition of the miR156-Overexpressed Arabidopsis

The precursor of sugarcane *miR156* was constructed in the overexpression vector pCAMBIA1301 and transformed into *Agrobacterium tumefacines* strain EHA105 [58]. Transformation of pCAMBIA *miR156* into *Arabidopsis* was performed according to a previously described method [27,59]. T3 transgenic *Arabidopsis* homozygotes were obtained and used for the subsequent experiments.

### 4.5. Low N Treatment of the miR156-Overexpressed Arabidopsis

MS medium containing 1/2 N (low N treatment, LN) was used, pH was adjusted to 5.8, and agar powder was added to 4.5 g/L. After disinfection, wild-type *Arabidopsis* seeds and homozygous *miR156*-overexpressed *Arabidopsis* seeds were sowed on the prepared plate. Each plate was a biological repeat, and three biological repeats were set for each transgenic line. The plates were wrapped with tin foil, treated for 3 days in the dark at 4 °C, and transferred to the incubator. After 20 days of cultivation (22 °C, 75% relative humidity, 16 h photoperiod), the whole plant samples were collected. Some fresh plant samples were used to determine the activities of key enzymes in N metabolism, and some plant samples were frozen in liquid N and stored in a −80 °C refrigerator for qRT-PCR detection of miRNAs and genes. Plant roots under low N treatment were analyzed by root analyzer (WinRHIZO system, Regent Instruments, Québec, Canada).

### 4.6. Physiological Changes and qRT-PCR Detection of the miR156-Overexpressed Arabidopsis under Low N Treatment

After 20 days of low N treatment, the activities of key N metabolism enzymes NR (nitrate reductase), NIR (nitrite reductase), GS (glutamine synthase), GOGAT (glutamate synthase) and GDH (glutamate dehydrogenase) in the *miR156*-overexpressed *Arabidopsis* were determined. At the molecular level, the gene expression levels of the *miR156* and key genes of N metabolism in the *miR156*-overexpressed *Arabidopsis* under low N treatment for 20 days were detected by qRT-PCR. The stem ring primer (5′-3′) of the *miR156* was CTCAACTGGTGTCGTGGAGTCGGCAATTCAGTTGAGGTGCTCAC. The qRT-PCR primers of the *miR156* and N metabolism key genes (*NRT1.1*, *AMT1.1*, *NR2*, *NR1*, *GS1*, *GDH1*, *FD-GOGAT*, *NADH-GOGAT*, *NIR1*) were shown in Table S5. The internal reference gene of the *miR156* and N metabolism key genes were *18S rRNA* [56] and *UBQ10* [60], respectively.

## 5. Conclusions

In the present study, the root miRNA database of the N-efficient sugarcane variety ROC22 under low N stress was constructed. According to GO and KEGG analysis, the predicted target genes of the differentially expressed miRNAs were mainly involved in the molecular functions of amylase synthase, mannosyltransferase, asparagine hydrolase, and in the biological processes of abiotic stimulation and hormone response. These target genes were mainly enriched in N metabolism, amino acid metabolism, carbohydrate metabolism, photosynthesis and hormone signal transduction pathways. Interestingly, there was a negative correlation between *miR168* and *SPX*, along with *miR396* and *acnA* under low N stress for 0–24 h. Moreover, the expression of *miR156* in the roots of ROC22 was significantly up-regulated under low N treatment and was negatively correlated with *SPL* and *P5CS* at stress for 3 h, 8 d, and 16 d. Furthermore, the length and surface area of roots in the *miR156*-overexpressed *Arabidopsis* were significantly higher than those of wild type under low N treatment, and the expression of key genes *NRT1.1*, *NR1*, *NIR1*, *GS* and the enzyme activity of NR and GS of N assimilation was all significantly higher than those of the wild type. It can thus be concluded that sugarcane *miR156* can enhance the nitrogen assimilation ability of *Arabidopsis* plants under a low N environment. In the future, *miR156* can be overexpressed or silenced in sugarcane, and the mechanism of *miR156* regulating NUE in sugarcane can be investigated to provide a theoretical basis for the development of sugarcane varieties with high NUE.

## Data Availability

Not applicable.

## Supplementary Materials

The following supporting information can be downloaded at: https://www.mdpi.com/article/10.3390/ijms232113187/s1.
