# Supplementary material for: Identification of Low-Nitrogen-Related miRNAs and Their Target Genes in Sugarcane and the Role of miR156 in Nitrogen Assimilation
Source: Int J Mol Sci. 2022 Oct 29;23(21):13187. doi: 10.3390/ijms232113187 (PMC9657601; doi:10.3390/ijms232113187)
Supplement: Supplementary file 1 [file ijms-23-13187-s001.zip › Figure S2.pdf]

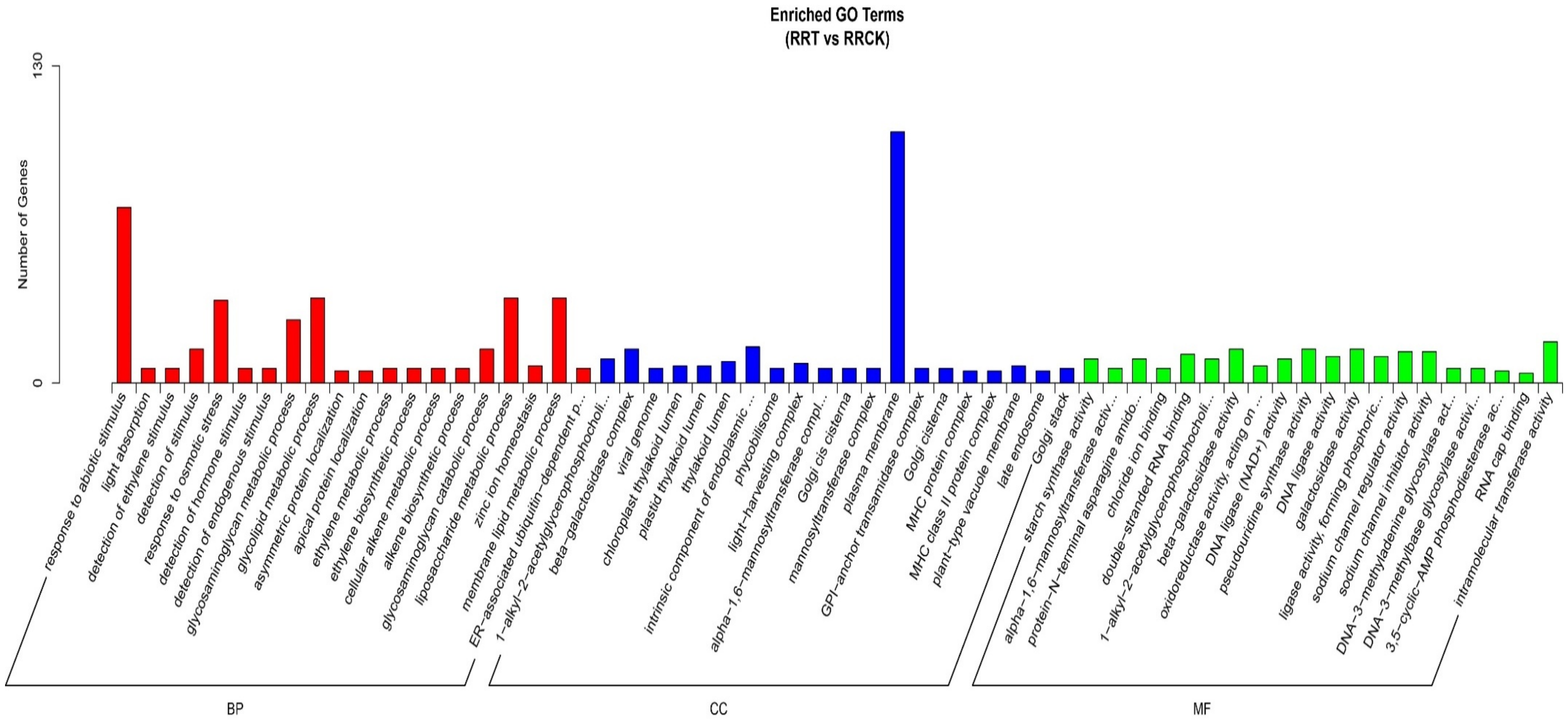

Figure S2. GO analysis of candidate target genes of differentially expressed miRNAs

Notes: BP, biological process; CC, cellular component; MF, molecular function; RRCK, the roots of ROC22 under low N stress for 0 h; RRT, the roots of ROC22 under low N stress for 3 h.
