# Supplementary material for: Identification of Low-Nitrogen-Related miRNAs and Their Target Genes in Sugarcane and the Role of miR156 in Nitrogen Assimilation
Source: Int J Mol Sci. 2022 Oct 29;23(21):13187. doi: 10.3390/ijms232113187 (PMC9657601; doi:10.3390/ijms232113187)
Supplement: Supplementary file 1 [file ijms-23-13187-s001.zip › Table S1.pdf]

Table S1 The quality assessment results for raw data

Sample	Read	Base/G	Q20/%	Q30/%	GC content/%
RRT1	12,524,008	0.626	98.96	98.05	52.12
RRT2	14,692,677	0.735	98.90	98.01	51.94
RRT3	14,238,990	0.712	98.98	98.08	52.44
RRCK1	13,028,628	0.651	98.73	97.76	51.05
RRCK2	12,442,229	0.622	98.76	97.82	51.63
RRCK3	12,835,710	0.642	98.76	97.82	51.39

Notes: Reads, the basic unit of sequence data; Bases, the number of sequences multiplied by its length; Q20/Q30, the ratio of bases with Phred (base mass value) greater than 20 or 30 to the total number of bases; GC content, the sum of G and C bases to the total number of bases. Error rate of all samples was 0.01%. RRCK, the roots of ROC22 under low N stress for 0 h; RRT, the roots of ROC22 under low N stress for 3 h.
