# Supplementary material for: Identification of Low-Nitrogen-Related miRNAs and Their Target Genes in Sugarcane and the Role of miR156 in Nitrogen Assimilation
Source: Int J Mol Sci. 2022 Oct 29;23(21):13187. doi: 10.3390/ijms232113187 (PMC9657601; doi:10.3390/ijms232113187)
Supplement: Supplementary file 1 [file ijms-23-13187-s001.zip › Table S2.pdf]

Table S2 The filtering information results of primary data

Sample	Total reads	Low quality reads	5’ adapter contamined	3’ adapter null or insert null	PloyA/T/G/C	Clean reads
RRT1	12,524,008 (100.00%)	15,868 (0.13%)	13,645 (0.11%)	319,935 (2.55%)	25,887 (0.21%)	12,148,671 (97.00%)
RRT2	14,692,677 (100.00%)	24,429 (0.17%)	9,825 (0.07%)	218,573 (1.49%)	42,021 (0.29%)	14,397,828 (97.99%)
RRT3	14,238,990 (100.00%)	17,138 (0.12%)	17,844 (0.13%)	119,877 (0.84%)	24,257 (0.17%)	14,059,874 (98.74%)
RRCK1	13,028,628 (100.00%)	26,444 (0.20%)	9,237 (0.07%)	135,379 (1.04%)	37,550 (0.29%)	12,820,003 (98.40%)
RRCK2	12,442,229 (100.00%)	23,684 (0.19%)	6,442 (0.05%)	563,885 (4.53%)	31,589 (0.25%)	11,816,613 (94.97%)
RRCK3	12,835,710 (100.00%)	23,915 (0.19%)	7,651 (0.06%)	224,149 (1.75%)	38,741 (0.30%)	12,541,239 (97.71%)

Notes: total reads, the total number of sequential data; low quality reads, the ratio of the number of reads removed due to low quality to the total number of raw reads; 5 adapter contamine, the ratio of the number of reads removed due to the inclusion of 5'joints to the total number of raw reads; 3 adapter null or insert null, the ratio of the number of reads removed due to the absence of 3' joints to the total number of raw reads; ployA/T/G/C, the ratio of the number of reads removed because of ployA/T/G/C to the total number of raw reads; clean reads, the ratio of the number of clean reads to the total number of raw reads. RRCK, the roots of ROC22 under low N stress for 0 h; RRT, the roots of ROC22 under low N stress for 3 h.
