# Supplementary material for: Identification of Low-Nitrogen-Related miRNAs and Their Target Genes in Sugarcane and the Role of miR156 in Nitrogen Assimilation
Source: Int J Mol Sci. 2022 Oct 29;23(21):13187. doi: 10.3390/ijms232113187 (PMC9657601; doi:10.3390/ijms232113187)
Supplement: Supplementary file 1 [file ijms-23-13187-s001.zip › Table S3.pdf]

Table S3 The sequences and stem loop primers of miRNAs in sugarcane

Group ID	MiRNA	Sequnce	Stem loop primer
1	*miR168a*	TCGCTTGGTGCAGATCGGGAC	CTCAACTGGTGTCGTGGAGTCGGCAATTCAGTTGAGGTCCCGAT
2	*novel-158*	AGGGATGGTATGCACTGAAGC	CTCAACTGGTGTCGTGGAGTCGGCAATTCAGTTGAGGCTTCAGT
3	*novel-57*	TACAACCGGCTTAGACACAAGA	CTCAACTGGTGTCGTGGAGTCGGCAATTCAGTTGAGTCTTGTGT
4	*miR393*	CTCCAAAGGGATCGCATTGAT	CTCAACTGGTGTCGTGGAGTCGGCAATTCAGTTGAGATCAATGC
5	*novel-215*	ACTCTGTAGTACGTAGTATGTAGG	CTCAACTGGTGTCGTGGAGTCGGCAATTCAGTTGAGCCTACATA
6	*miR408a*	CTGCACTGCCTCTTCCCTGGC	CTCAACTGGTGTCGTGGAGTCGGCAATTCAGTTGAGGCCAGGGA
7	*miR396*	TCCACAGGCTTTCTTGAACTG	CTCAACTGGTGTCGTGGAGTCGGCAATTCAGTTGAGCAGTTCAA
8	*miR156*	TGACAGAAGAGAGTGAGCAC	CTCAACTGGTGTCGTGGAGTCGGCAATTCAGTTGAGGTGCTCAC
9	*miR167*	TGAAGCTGCCAGCATGATCTG	CTCAACTGGTGTCGTGGAGTCGGCAATTCAGTTGAGCAGATCAT
10	*miR408e*	CTGCACTGACTCTTCCCTGGC	CTCAACTGGTGTCGTGGAGTCGGCAATTCAGTTGAGGCCAGGGA
