# Supplementary material for: Identification of Low-Nitrogen-Related miRNAs and Their Target Genes in Sugarcane and the Role of miR156 in Nitrogen Assimilation
Source: Int J Mol Sci. 2022 Oct 29;23(21):13187. doi: 10.3390/ijms232113187 (PMC9657601; doi:10.3390/ijms232113187)
Supplement: Supplementary file 1 [file ijms-23-13187-s001.zip › Table S4.pdf]

Table S4 The qRT-PCR primers for miRNAs and their potential target genes in sugarcane

Group ID	MiRNA/target	Gene ID	Forward primer	Reverse primer
1	*P5CS*	sample|Cluster-9046.62773	ACGGACAGTCTGCTTGACAG	TCGTGTCGTTGGTGAGACTG
2	*acnA*	sample|Cluster-1234.10611	TGGCTGCCCATGAAAGTCAA	GTCGGGTTCAAGGGTTTTGC
3	*SPX*	sample|Cluster-9046.203583	GCGCTCAACTACACTGGACT	TCGCTGTCGCTGTCTTCTTT
4	*GS*	sample|Cluster-9046.93347	GTGGCCGTCTGTTCCCATAA	GCAAAGACCCCAACAAGCTG
5	*AUX/IAA*	sample|Cluster-9046.46359	TGACGAAGTTGATGGGAGGC	CCCACGGAACATCTCCAACA
6	*TIR1*	sample|Cluster-82409.0	CGAAGGGATGCTCCACCTTT	GGTATCCCAGTGCAACACCA
7	*ACO1*	sample|Cluster-9046.137637	GGAGAGCACCTTCTTCGTCC	GATGCGAACTGCTTCGTCAC
8	*NiFU*	sample|Cluster-9046.167793	AGCGATGGACTCAACTGCAA	GCTACAAGGAGCATGTGGGT
9	*ARF*	sample|Cluster-9046.156023	CTGGGCCTCAAGGCCAAATA	TGACCAAGTGAACCGACCAC
10	*SPL1*	sample|Cluster-9046.200116	GTAGCCGATGGGGACAACAA	CGTCAACCTGACAAGACGGA
11	*GRF*	sample|Cluster-9046.236180	GGCGGTTCAATGTGGGAGTA	CGAGGGGCTTCACCATTGAT
12	*AGO*	sample|Cluster-9046.39783	CACAATGTCACGCAGCACAA	AATTCGTGGTCACCTTGCCT
13	*PC*	sample|Cluster-9046.239128	GAAGATGGGGACGACGAAGG	GTTAGGCTATCTCCCGGTGC
14	*GADPH*		CACGGCCACTGGAAGCA	TCCTCAGGGTTCCTGATGCC
15	*miR168a*		ACACTCCAGCTGGGTCGCTTGGTGCAGAT	TGGTGTCGTGGAGTCG
16	*novel-158*		ACACTCCAGCTGGGAGGGATGGTATGCAC	TGGTGTCGTGGAGTCG
17	*novel-57*		ACACTCCAGCTGGGTACAACCGGCTTAGAC	TGGTGTCGTGGAGTCG
18	*miR393*		ACACTCCAGCTGGGCTCCAAAGGGATCGC	TGGTGTCGTGGAGTCG
19	*novel-215*		ACACTCCAGCTGGGACTCTGTAGTACGTAGTA	TGGTGTCGTGGAGTCG
20	*miR408a*		ACACTCCAGCTGGGCTGCACTGCCTCTTC	TGGTGTCGTGGAGTCG
21	*miR396*		ACACTCCAGCTGGGTCCACAGGCTTTCTT	TGGTGTCGTGGAGTCG
22	*miR156*		ACACTCCAGCTGGGTGACAGAAGAGAGT	TGGTGTCGTGGAGTCG
23	*miR167*		ACACTCCAGCTGGGTGAAGCTGCCAGCAT	TGGTGTCGTGGAGTCG
24	*miR408e*		ACACTCCAGCTGGGCTGCACTGACTCTTC	TGGTGTCGTGGAGTCG

25	*18SrRNA*	CTACGTCCCTGCCCTTTGTACA	ACACTTCACCGGACCATTCAA
