# Supplementary material for: Identification of Low-Nitrogen-Related miRNAs and Their Target Genes in Sugarcane and the Role of miR156 in Nitrogen Assimilation
Source: Int J Mol Sci. 2022 Oct 29;23(21):13187. doi: 10.3390/ijms232113187 (PMC9657601; doi:10.3390/ijms232113187)
Supplement: Supplementary file 1 [file ijms-23-13187-s001.zip › Table S5.pdf]

Table S5 The qRT-PCR primers for *miR156* and N metabolism key genes in transgenic *Arabidopsis*

Group ID	MiRNA and gene	Forward primer (5'-3')	Reverse primer (5'-3')
1	*miR156*	ACACTCCAGCTGGGTGACAGAAGAGAGT	TGGTGTCGTGGAGTCG
2	*18S rRNA*	CTACGTCCCTGCCCTTTGTACA	ACACTTCACCGGACCATTCAA
3	*NRT1.1*	TAAGGGATCAGGAAGCGGGA	TGGAACGGTCCAATGTCTCG
4	*AMT1.1*	ATCGCTGAACGGACACAGTT	GCCCATCCATCAACTGACCA
5	*NR2*	CCACGTCCCTAAAGCCCAAT	TCTGTTCCTTACGGCGGTTC
6	*NR1*	GAAACTAGCAATGCTCGCCG	ATCCTCGGTTCTGTTTGCGT
7	*GS1*	CTAGTGAGCTCATGCCAGGG	TCAGGAGCAGGCTTTCCTTG
8	*GDH1*	GCTAGGCTTCTTGGTCTGGA	GGGACCTCTTGCATTGTCGT
9	*FD-GOGAT*	AGCTTTAAGACCTCAGGTTGCT	GTTCCTTGGCCCAGACGTTA
10	*NADH-GOGAT*	AGGACACACAGGCGTAAAGG	AGGGCAAAAACAAGAAGCAGG
11	*NIR1*	GTCTTAGCTTCGTGGGGCTT	GGCTACCGGTACAAGCAACT
12	*UBQ10*	AGAAGTTCAATGTTTCGTTTCATGTAA	GAACGGAAACATAGTAGAACACTTATTCA
